# In Vitro and In Vivo Antipsoriatic Efficacy of Protected and Unprotected Sugar–Zinc Phthalocyanine Conjugates

**DOI:** 10.3390/pharmaceutics16060838

**Published:** 2024-06-20

**Authors:** Sebastian Makuch, Piotr Kupczyk, Marta Woźniak, Alicja Makarec, Maja Lipińska, Magdalena Klyta, Joanna Sulecka-Zadka, Wiesław Szeja, Mariachiara Gani, Valentina Rapozzi, Piotr Ziółkowski, Piotr Smoleński

**Affiliations:** 1 Department of Clinical and Experimental Pathology, Wroclaw Medical University, 50-368 Wroclaw, Poland; piotr.kupczyk@umw.edu.pl (P.K.); marta.wozniak@umw.edu.pl (M.W.); alicja.makarec@umw.edu.pl (A.M.); piotr.ziolkowski@umw.edu.pl (P.Z.); 2 Experimental Animal Facility, Wroclaw Medical University, 50-368 Wroclaw, Poland; maja.lipinska@umw.edu.pl (M.L.); magdalena.klyta@umw.edu.pl (M.K.); 3 Department of Pharmacology and Toxicology, Faculty of Veterinary Medicine, Wroclaw University of Environmental and Life Sciences, 50-375 Wroclaw, Poland; joanna.sulecka-zadka@upwr.edu.pl; 4 Department of Organic Chemistry, Bioorganic Chemistry and Biotechnology, Silesian University of Technology, B. Krzywoustego 4, 44-100 Gliwice, Poland; wieslaw.szeja@adres.pl; 5 Department of Medicine, Laboratory of Biochemistry, P.le Kolbe 4, 33100 Udine, Italy; gani.mariachiara@spes.uniud.it (M.G.); valentina.rapozzi@uniud.it (V.R.); 6 Faculty of Chemistry, University of Wrocław, F. Joliot-Curie 14, 50-383 Wroclaw, Poland

**Keywords:** zinc phthalocyanine, sugar conjugates, psoriasis, cytokines, drug uptake

## Abstract

Psoriasis, a chronic immune-mediated skin disorder affecting over 125 million people globally, is characterized by abnormal keratinocyte proliferation and immune cell infiltration. Photodynamic therapy (PDT) remains underutilized in the treatment of psoriasis despite its potential as a promising and effective therapeutic approach. This study aimed to explore the efficacy of zinc phthalocyanine (ZnPc) and its sugar conjugates as potential antipsoriatic agents. We successfully synthesized protected and unprotected sugar-conjugated zinc phthalocyanines and evaluated their potential against cytokine-stimulated HaCaT keratinocytes, as well as an established IMQ psoriasis-like in vivo model. Tetrasubstituted protected glucose–ZnPc (Glu-4-ZnPc-P) demonstrated superior phototoxicity (IC50 = 2.55 µM) compared to unprotected glucose conjugate (IC50 = 22.7 µM), protected galactose–ZnPc (IC50 = 7.13 µM), and free ZnPc in cytokine-stimulated HaCaT cells (IC50 = 5.84 µM). Cellular uptake analysis revealed that IL-17A, a cytokine that plays a central role in the pathogenesis of psoriasis, enhanced unprotected Glu-4-ZnPc uptake by 56.3%, while GLUT1 inhibitor BAY-876 reduced its accumulation by 23.8%. Intracellular ROS generation following Glu-4-ZnPc-P-PDT was significantly increased after stimulation with IL-17A, correlating with in vitro photocytotoxicity. In vivo PDT using Glu-4-ZnPc-P exhibited significant improvement in Psoriasis Area and Severity Index (PASI), inhibiting splenomegaly and restoring normal skin morphology. This study highlights sugar-conjugated zinc phthalocyanines as potential candidates for targeted PDT in psoriasis, providing a basis for further clinical investigations.

## 1. Introduction

Psoriasis is an immune-mediated, chronic, and noncontagious skin disease. It was estimated that the prevalence varies from 0.14% to 5.32% of the population [1], affecting approximately 125 million people worldwide [2]. It is characterized by abnormal proliferation of keratinocytes, infiltration of immune cells, and epidermal hyperplasia of dermal vessels. The most common form is plaque psoriasis, which can appear as raised areas of inflamed skin covered with red and silvery scales [3,4]. The pathogenesis of psoriasis is a complex and multi-factorial process that can be mediated by proinflammatory cytokines, such as IL-6, IL-17, and IL-23 [2,5].

One of the recommended treatments from the guidelines of care for the management of psoriatic skin is phototherapy [6]. Studies showed promising effects of photodynamic therapy (PDT) for psoriasis, a form of phototherapy in which inappropriate cells are selectively destroyed by reactive oxygen species (ROS) [7]. PDT is based on the interaction between photosensitizer, light at a specific wavelength, and oxygen [8]. Originally, PDT was used to treat cutaneous disorders and later evolved as antitumoral therapy [9]. Many photosensitizers for PDT are widely researched, such as first-generation photosensitizer aminolevulinic acid (ALA). However, patients who received ALA-PDT frequently experienced intense pain and burning sensations, which in many cases were intolerable for them [10]. This underscores the need to explore second-generation photosensitizers in scientific investigations [11].

Phthalocyanines (Ps), developed over a span of 75 years primarily as blue and green pigments, have emerged as pivotal organic dyes [11]. In recent times, their applications have expanded to include optoelectronics and photoelectronics, notably in LCD displays, along with serving as a photoconductor in laser printers [11]. The basis for exploring additional applications lies in the relative simplicity of synthesis, coupled with the extensive potential for tailoring properties through the strategic selection of molecular structures [12]. The photophysical properties of phthalocyanine dyes are significantly influenced by the presence and nature of the coordinated central metal. Phthalocyanines with closed-shell, diamagnetic ions such as Zn^2+^, Al^3+^, and Ga^3+^ exhibit both high triplet yields and extended excited triplet state lifetimes [13]. Consequently, these macrocycles, particularly those involving zinc, demonstrate favorable photochemical and photodynamic characteristics attributed to their efficient generation of reactive oxygen species. Owing to their low toxicity, these compounds have proven to be a promising class of photosensitizers, finding application in biological and medical research, particularly in photodynamic therapy [14]. They are characterized by long excitation wavelength (670 nm), especially compared to ultraviolet light therapy, which allows deeper penetration into the skin [15,16]. Moreover, phthalocyanine displays better safety and effectiveness [15]. Studies have demonstrated a positive effect of zinc phthalocyanine (ZnPc) on melanoma cancer [17], as well as a photosensitizer for PDT of esophageal cancer [18].

However, there are limited data on the effectiveness of second-generation photosensitizers in the treatment of psoriasis [19]. Liu et al. performed an in vivo study showing that α-(8-quinolinoxy) zinc phthalocyanine (ZnPc-F7)-mediated PDT reduced the psoriatic symptoms. The metallophthalocyanine compound was characterized by suitable solubility, a strong effect, and low toxicity [20]. Another study introduced an amphiphilic zinc phthalocyanine polymer conjugate (ZPB) with a core-shell nanostructure, demonstrating a positive effect of photodynamic therapy against psoriasis in a guinea pig model [15].

It has been proven that glucose metabolism is essential for the proliferation of keratinocytes, hence the overexpression of glucose transporters, especially GLUT1 [21,22]. A study conducted by Zhang et al. showed that overexpressed GLUT1 transporters and increased glucose uptake may be the new potential target for the treatment of psoriasis and other hyperproliferative skin diseases [21]. The aim of our study was to confirm if sugar conjugation with zinc phthalocyanine leads to enhanced uptake of the conjugated drug through GLUT1 by rapidly proliferating HaCaT keratinocytes and verify the antipsoriatic activity of the conjugates in vivo.

## 2. Materials and Methods

### 2.1. Materials and Analytical Methods

All standard chemicals and solvents were obtained from commercial suppliers (zinc acetate dehydrate, 1,2:3,4-di-*O*-isopropylidene-α-D-galactopyranose and 1,2:5,6-di-*O*-isopropylidene-α-D-glucofuranose—Sigma-Aldrich, St. Louis, MO, USA; 1,8-diazabicyclo-[5.4.0]undec-7-ene—Thermo Scientific, Waltham, MA, USA; and 4-nitrophthalonitrile—TCI Chemicals, Tokyo, Japan). Free zinc phthalocyanine (ZnPc) was obtained from TCI Chemicals, Tokyo, Japan.

Bruker IFS 1113v (Ettlingen, Germany) was used to measure the FT-IR spectra (range of 4000–400 cm^−1^) abbreviations: vs, very strong; s, strong; m, medium; w, weak; br., broad). NMR spectra were recorded in DMSO-*d*_6_ and DMF-*d*_7_ solvents using a Bruker 600 AMX spectrometer (Bruker BioSpin MRI GmbH, Ettlingen, Germany) at ambient temperature (abbreviations: br, broad, m, multiplet). ^1^H chemical shifts (*δ*) are expressed in ppm relative to Si(Me)_4_. The Elemental Analyser Vario ELCube (Elementar Analysen Systeme GmbH, Langenselbold, Germany) was used for the determination of C, H, and N contents (Laboratory of Elemental Analysis at Faculty of Chemistry, University of Wrocław). Luminescence spectra, as well as at 300 K, were recorded on an FSL980 spectrofluorometer (Edinburgh Instruments, Livingston, UK). The H_2_O solution (10^−3^ M) of (**Glu-4-ZnPc**) was used for the MALDI-TOF-MS experiment that was carried out on a JEOL JMS-S3000 SpiralTOF™-plus Ultra-High Mass Resolution MALDI-TOFM mass spectrometer (JEOL Ltd., Tokyo, Japan) at the Faculty of Chemistry, University of Wrocław). HPLC analyses were performed using the HPLC system Dionex with a UV–Vis detector (Dionex Corporation, Sunnyvale, CA, USA). Column: Phenomenex Aeris PEPTIDE XB-C18, 250 × 4.6 mm, particle diameter 5 µm (Phenomenex, Torrance, CA, USA). Mobile phase: gradient from 0% B in A to 80% B in A in 30 min, then 100% B for 2 min and 0% B for 5 min. A: water containing 0.1% TFA. B: acetonitrile/water (80/20, *v*/*v*) containing 0.1% TFA (TFA: trifluoroacetic acid). All reagents were HPLC-gradient grade.

4-(1,2:5,6-Di-*O*-isopropylidene-α-D-glucofuranosyl)phthalonitrile, 4-(1,2:3,4-Di-*O*-isopropylidene-α-D-galactopyranosyl)phthalonitrile, [1(4),8(11),15(18),22(25)-tetrakis(1,2:5,6-di-*O*-isopropylidene-α-D-glucofuranosyl)phthalocyaninato]zinc(II) (**Glu-4-ZnPc-P**) and [1(4),8(11),15(18),22(25)-tetrakis(1,2:3,4-di-O-isopropylidene-α-D-galactopyranosyl)phthalocyaninato]zinc(II) (**Gal-4-ZnPc-P**) were synthesized according to the reported procedure [23], while compound (**Glu-4-ZnPc**) based on procedure described for synthesis of [1,2,3,4-Tetrakis(α/β-D-galactopyranos-6-yl)-phthalocyaninato]zinc(II) by Cavaleiro et al. [24]. All of the compounds were dissolved in DMSO, as **Glu-4-ZnPc-P, Gal-4-ZnPc-P,** and **ZnPc** are insoluble in water.

### 2.2. Synthesis of [1(4),8(11),15(18),22(25)-Tetrakis(1,2:5,6-tetrahydroxy-α-D-glucofuranosyl)-phthalocyaninato]zinc(II) (Glu-4-ZnPc)

100 mg (62.1 µmol) of [1(4),8(11),15(18),22(25)-Tetrakis(1,2:5,6-di-*O*-isopropylidene-α-D-glucofuranosyl)phthalocyaninato]zinc(II) was dissolved in 25 mL of TFA/water mixture (*v*:*v*, 9:1). The dark-green mixture was stirred in the absence of light for four hours at room temperature under nitrogen. The volatile components were removed through evaporation under vacuum, and the remaining residue was dissolved in a solution of 1% acetic acid in water. The mixture was stirred at ambient conditions for one day, after which it was again dried under vacuum. It was then washed twice with 10 mL of chloroform and finally dried in vacuo. **Glu-4-ZnPc** was obtained as a dark-green solid with a yield of 90%, based on **Glu-4-ZnPc-P**. IR (KBr, cm^−1^): 3428 (br, vs), 2919 (m), 2851 (m), 2365 (w), 2333 (w) 1678 (br, s), 1612 (br, s), 1485 (m), 1402 (m), 1336 (m), 1286 (m), 1228 (s), 1206 (s), 1130 (m) 1084 (m), 1047 (m), 944 (w), 840 (w) 801 (w), 745 (m), 724 (m), 669 (w). ^1^H NMR (600.15 MHz, DMSO-*d*_6_): *δ*, ppm, 9.30–9.25 (m, 4 H, Pc-*H_α_*), 9.12–9.07 (m, 4 H, Pc-*H_α_*), 7.98–7.94 (m, 4 H, Pc-*H_β_*). 5.55–3.25 (set of multiplets, 44 H, sugar-*H,* partially overlapped in the range of 3.5–3.0 ppm). ^1^H NMR (600.15 MHz, DMF-*d*_7_): *δ*, ppm, 9.33–9.29 (m, 4 H, Pc-*H_α_*), 9.25–9.13 (m, 4 H, Pc-*H_α_*), 8.15–8.00 (m, 4 H, Pc-*H_β_*, partially overlapped by DMF). 5.76–3.65 (set of multiplets, 44 H, sugar-*H*, partially overlapped in the range of 3.75–2.8 ppm). C_56_H_56_N_8_O_24_Zn (**Glu-4-ZnPc**) (MW 1290.5 + 8H_2_O): C, 46.88; N, 7.81; H, 5.06. Found: C, 46.46; N, 7.61; H, 5.48. MALDI-TOF-MS: *m*/*z* 1289.275 (calcd. for C_56_H_56_N_8_O_24_Zn, [M]^+^ 1289.49). The purity of the **Glu-4-ZnPc** was determined based on HPLC measurement and found to be around 95% (Figures S6 and S7).

### 2.3. Cell Culture

The human keratinocytes cell line HaCaT was obtained from Cell Lines Service (CLS Cell Lines Service, Eppelheim, Germany; 300493) and maintained in Dulbecco’s modified Eagle’s medium (DMEM, high glucose) supplemented with 10% *v*/*v* fetal bovine serum (FBS), 1% *v*/*v* antibiotics (100 U/mL penicillin, 100 µg/mL streptomycin), 1% *v*/*v* L-Glutamine (2 mM) at 37 °C and 5% CO_2_ in a humidified incubator. Cell culture reagents were purchased from Gibco (Thermo Fisher Scientific, Waltham, MA, USA). The culture medium was renewed every 3 days. For the experiments, HaCaT cells were incubated with exogenous cytokines IL-6, IL-17A, and IL-23 (all from PeproTech, Cranbury, NJ, USA) for 24 h at a final concentration of 100 ng/mL each.

### 2.4. Dark Cytotoxicity and Cell Proliferation Assay

Cell proliferation was evaluated by utilizing the conversion of the yellow tetrazolium salt (MTT) into violet formazan insoluble crystals in the mitochondria of active cells. To assess the cytotoxicity of zinc phthalocyanine–sugar conjugates, HaCaT cells were seeded at a density of 1 × 10^4^ per well in a 96-well plate and stimulated the following day with IL-6, IL-17A, and IL-23 for 24 h at a final concentration of 100 ng/mL each (PeproTech, Cranbury, NJ, USA). The next day, phthalocyanine solutions dissolved in DMSO and diluted in a culture medium at concentrations from 0.1 to 10 μM were added for 4 h and kept in the dark. Then, cells were irradiated at a fluence of 0.9 J/cm^2^ using a halogen lamp (Penta Lamps, Teclas, Lugano, Switzerland). The following day, the photosensitizer solutions were removed from each well, and an MTT assay was performed. After 4 h of incubation with 0.5% MTT solution, the medium was removed, and the dye was dissolved by dimethyl sulfoxide (DMSO, Sigma-Aldrich, Munich, Germany), creating the color, whose intensity is proportional to the viable cells. The optical absorbance was measured at 490 nm using a BioTek ELX800 multi-well reader (BioTek, Winooski, VT, USA). Dark cytotoxicity was evaluated under the same conditions but without light irradiation. Each compound concentration was tested in four replicates and repeated at least three times. Based on the results, the dose of 2.5 µM was selected for the rest of the experiments for comparison, as higher concentrations were displaying dark cytotoxicity.

### 2.5. Cellular Uptake by Flow Cytometry

The cellular uptake of investigated photosensitizers was determined by using flow cytometry and quantified based on phthalocyanines’ red fluorescence. HaCaT cells were seeded on a 12-well culture plate at a density of 15 × 10^4^ per well. The next day, cells were incubated with 100 ng/mL of exogenous cytokines (IL-6, IL-17A, or IL-23) for 24 h or BAY-867, a selective GLUT1 inhibitor, for 2 h before drug administration, at a concentration of 1 mM. Then, cells were washed 3× with phosphate-buffered saline (PBS) and incubated with phthalocyanines at 2.5 µM for 24 h in darkness. After incubation, cells were washed two times with PBS and harvested for analysis. Then, cells were collected by centrifugation, resuspended in 200 μL of PBS, and analyzed by flow cytometry using Cytoflex (Beckman Coulter, Brea, CA, USA). The detection of the fluorescent signal originating from phthalocyanines involved the use of a 638 nm laser for excitation and a 660/20 nm emission filter and was detected using the APC channel in the logarithmic scale. Each test evaluated a total of ten thousand cells. Data were subjected to analysis using CytExpert 2.0 software (Beckman Coulter, Brea, CA, USA) and presented as mean fluorescence.

### 2.6. Fluorescent Microscopy

HaCaT cells were cultured at a density of 2 × 10^4^ cells per well on Millicell^®^ EZ Slide (Merck KGaA, Darmstadt, Germany) at 37 °C and allowed to incubate for 24 h. Subsequently, phthalocyanine–sugar conjugates were administrated, with a final concentration of 2.5 µM. Following 24 h incubation, cells were washed with PBS to eliminate unbound phthalocyanines, and a serum-free medium containing MitoTracker Green (40 nM) for 30 min or LysoTracker Green (75 nM) for 10 min was added, then washed 3 times with PBS, and placed in fresh medium. Cell imaging was performed using a fluorescent microscope Axio Observer 7 (Zeiss, Oberkochen, Germany). The argon-ion laser light (wavelength 488 nm or 633 nm) excited the fluorescence of MitoTracker Green, LysoTracker Green, or phthalocyanine–sugar conjugates in the cells on the chamber slides, while emitted fluorescence was filtered using barrier filters (517 nm or 664 nm, respectively). Colocalization of the phthalocyanines with mitochondria and lysosomes was measured using Just Another Colocalization Plugin (JACoP) on Fiji (ImageJ distribution, version 1.54g, National Institutes of Health, Bethesda, MD, USA) to obtain Pearson’s correlation coefficients [25].

Throughout the entire imaging experiment, parameters such as laser-line intensity and photometric gain remained constant.

### 2.7. ROS Generation In Vitro after PDT

ROS levels were assessed by monitoring the intracellular peroxide-dependent oxidation of DCFH-DA, resulting in the formation of the fluorescent compound 2,7-dichlorofluorescein (DCF). HaCaT cells were seeded onto a 96-well plate at a density of 15 × 10^3^ cells per well and cultured overnight. The next day, cells were stimulated with 100 ng/mL of exogenous cytokines (IL-6, IL-17A, or IL-23). Subsequently, fresh medium containing the studied phthalocyanines (2.5 µM) was introduced, and the cells were incubated for 24 h in darkness. After three washes with PBS, 50 µL of DCFH-DA (10 µM) was added, and the cells were further incubated for 30 min. Following the removal of the medium and three additional PBS washes, the cells were exposed to laser light (670 nm) with an illumination dose of 0.9 J/cm^2^. Following illumination, the cells were lysed with 1% SDS (100 µL) for 10 min on a table concentrator, and the DCF fluorescence was quantified using a Bio-Tek microplate reader (Corning Incorporated, New York, NY, USA) with excitation/emission wavelengths of 488/525 nm.

### 2.8. Animal Experiments

C57BL/6 mice, aged 8 to 11 weeks, with a body weight range of 15 g to 29 g, received a daily topical dose of 62.5 mg of 5% imiquimod (IMQ) (Aldara; Meda AB, Solna, Sweden) on a shaved skin area measuring 3 cm × 4 cm on the back for seven days. A total of 60 mice (half male and half female) were divided into six groups: (a) control; (b) IMQ treated with DMSO (0.625 mL/kg, which is 1.25%); (c) light exposure control; (d) Glu-4-ZnPc-P-PDT low dose (0.30 mg/kg), Glu-4-ZnPc-P-PDT medium dose (0.60 mg/kg), and Glu-4-ZnPc-P-PDT high dose (1.20 mg/kg). Each experimental group consisted of 10 mice, a number determined by statistical methods (sample size calculation, power analysis) to ensure that the anticipated minimum animal numbers for each group are sufficient for a reliable estimation of the efficacy of the applied therapy. After IMQ application, Glu-4-ZnPc-P dissolved in DMSO was administered intravenously via the tail vein using 0.5 mm syringes with a needle diameter not exceeding 25–30 G at a volume of 25 µL per body mass (20 g)—resulting in a final DMSO concentration of 0.625 mL/kg–1.25%. A total of 4 h after the administration of Glu-4-ZnPc-P, the mouse skin was illuminated with a red laser at an intensity of 15 J/cm^2^ for 7 min. The output power, spot diameter, and irradiation time were set at 1.00 W, 6 cm, and 420 s, respectively. Every day, from the first IMQ application until 7 days after illumination, the Psoriasis Area and Severity Index (PASI) was assessed. PASI index was used to evaluate erythema, plaque thickness, and scaling, all on a scale from 0 (no changes) to 4 (very severe changes), to a total score of 12. After IMQ treatment and 7 days following irradiation, the skin, spleen, liver, and blood samples were collected for further experiments. The blood for the ELISA experiment was collected by cardiac puncture.

The study was approved by the Local Ethics Committee on Animal Experiments (LKE) in Wroclaw, Poland (No. 035/2023).

### 2.9. Histology

Mice skin fragments were excised and prepared for fixation in 10% buffered formalin (Chempur, Piekary Śląskie, Poland) for 24 h. On the following day, the skins underwent standard dehydration processing in an escalating ethanol solution (50%, 70%, 80%, 95%, and 100%) for 10 min each, followed by two incubations with xylene for 10 min and subsequent paraffin embedding. The formalin-fixed paraffin-embedded (FFPE) tissue blocks were sliced into 5 μm sections using a microtome (Leica, Wetzlar, Germany). These sections were subjected to the routine hematoxylin and eosin (H&E) staining protocol. The confirmation of the psoriasis-like skin inflammation model was performed independently by two histopathologists. The specimens were analyzed for epidermal thickness, determination of the intensity and type of inflammatory cell infiltration in all skin compartments using an optical microscope Olympus BX53 with a digital camera ColorView IIIu (Olympus, Toyko, Japan), and cell^A picture analysis software (Olympus Soft Imaging Solution GmbH, Münster, Germany).

### 2.10. Determination of Serum Cytokine Levels (ELISA)

The serum levels of IL-6 (cat. no. KMC0061), IL-17A (cat. no. BMS6001), and IL-23 (cat. no. BMS6017) were determined using sandwich ELISA kits obtained from GIBCO^®^ Invitrogen GmbH (Thermo Fisher Scientific, Waltham, MA, USA) according to the manufacturer’s protocol.

### 2.11. Statistical Analysis

Statistical analyses of in vitro and in vivo data were performed using Prism 8.0.1 (GraphPad Software Inc., San Diego, CA, USA) by applying the unpaired *t*-test. The in vitro results were presented as the mean ± SD from three independent experiments. Sample size calculation and power analysis were used to determine the number of animals; *p* values lower than 0.05 were considered statistically significant.

## 3. Results and Discussion

**Figure 1:**
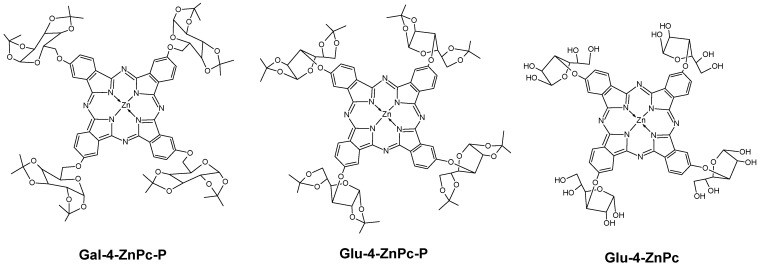
Structural formulas of glycosubstituted zinc phthalocyanines.

Synthetic methodology. The reaction of 4-nitrophthalonitrile with 1,2:5,6-di-O-isopropylidene-α-D-glucofuranose or 1,2:3,4-di-O-isopropylidene-α-D-galactopyranose in the presence of K_2_CO_3_ gave the corresponding glycosubstituted phthalonitriles, by Choi et al. procedure [23]. Self-cyclization of the precursors produced tetra-glycosylated zinc(II) phthalocyanines Glu-4-ZnPc-P and Gal-4-ZnPc-P (Figure 1). Additionally, a water-soluble derivative, [1(4),8(11),15(18),22(25)-tetrakis(1,2:5,6-tetrahydroxy-α-D-glucofuranosyl)phthalocyaninato]zinc(II) (Glu-4-ZnPc), can be obtained by deprotecting the hydroxyl groups of connected sugar molecules in the reaction of tetrakis(1,2:5,6-di-O-isopropylidene-α-D-glucofuranosyl)phthalocyaninato]zinc(II) (Glu-4-ZnPc-P) with a solution of TFA in water (*v*:*v*, 9:1) with high yield. The resulting product is a dark-green solid that is soluble and stable in the presence of air in polar solvents such as DMSO, water, and methanol. To remove the propylidene protections, an adapted procedure from Ribeiro et al. for a comparable class of compounds was employed [24].

The ^1^H NMR spectra of Glu-4-ZnPc-P in DMSO-*d*_6_ (Figure S2) and DMF-*d*_7_ (Figure S3) indicate deprotection of the carbohydrate groups as evidenced by the absence of signals from the isopropylidene protons. The analysis reveals three resonances centered between 9.30 and 7.94 ppm, which are due to the eight Pc-α and four Pc-β protons. The signals corresponding to the protons of the glycosylated moieties are found between 5.76 and 3.25 ppm and are partially overlapped by signals of water, especially in DMSO-*d_6_* solvent. Although some impurities are detected in ^1^H NMR spectra, the HPLC technique confirms the purity of Glu-4-ZnPc-P at approximately 95% (Figures S6 and S7). The MALDI-TOF-MS mass spectrum also confirms the molecular structure of the obtained water-soluble glycosylated derivative of phthalocyanine (Figures S4 and S5).

The emission spectra of **Glu-4-ZnPc** are shown in Figure S8, while Figure S9 presents the UV–Vis spectra of this compound in DMSO and water. The excitation spectrum in DMSO is well defined, indicating no intermolecular aggregation. The sharp Q band at 687 nm, which originates from the S_0_ → S_1_ transition, indicates that the compound exists as a monomeric species in the DMSO solution [26]. However, its optical properties differ significantly between water and DMSO. In water, the Q band intensity is much lower than in DMSO, suggesting that the molecules aggregate due to the cofacial arrangement of the Glu-4-ZnPc [24]. The B-band of the water solution is slightly shifted to a shorter wavelength compared to the spectrum in DMSO (340 and 357 nm for DMSO and water spectra, respectively). Additionally, the Q band in the case of the water solution is also blue-shifted and split into two absorptions at 642 nm and in the range of 660–680 nm.

### 3.1. Glu-4-ZnPc-P Shows Better Photocytotoxic Activity Compared to Glu-4-ZnPc, Gal-4-ZnPc-P, and ZnPc

**Figure 2:**
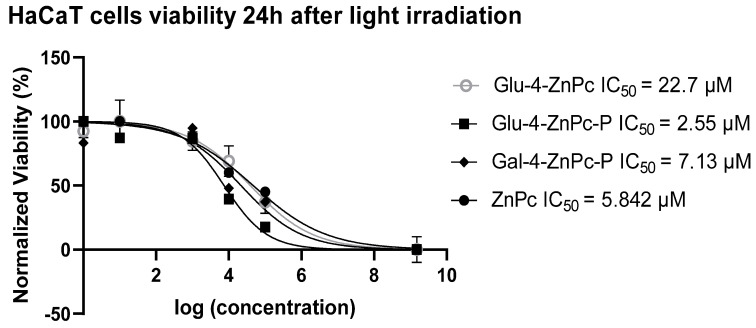
IC_50_ values of sugar-conjugated ZnPc compared to free ZnPc against HaCaT cells at a light dose of 0.9 J/cm^2^ 24 h after light irradiation.

**Figure 3:**
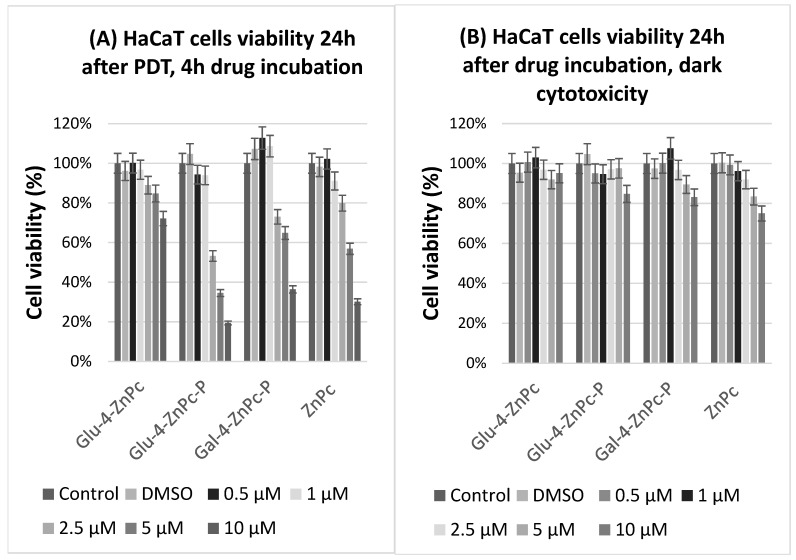
Cell viability of HaCaT cells stimulated with proinflammatory cytokines following 4 h or 24 h incubation with sugar–ZnPc conjugates or free ZnPc; (**A**) photocytotoxicity at 0.9 J/cm^2^; (**B**) dark cytotoxicity.

To evaluate the efficacy of sugar-conjugated zinc phthalocyanines, their dark and phototoxicity toward HaCaT cells stimulated with exogenous cytokines such as IL-6, IL-17A, and IL-23, which play a key role in the pathogenesis of psoriasis, was assessed using MTT assay. The photosensitizers were incubated with cells for 4 h in the dark and then irradiated with red light at a fluence of 0.9 J/cm^2^. The viability was tested 24 h after irradiation. Dark cytotoxicity was evaluated under the same conditions but without the red light irradiation (after 28 h in total). Glu-4-ZnPc-P showed the highest phototoxicity toward cytokine-stimulated HaCaT cells, with IC_50_ = 2.55 µM, compared to Glu-4-ZnPc (IC_50_ = 22.7 µM), Gal-4-ZnPc-P (IC_50_ = 7.13 µM), and ZnPc (IC_50_ = 5.842 µM) after 24 h (Figure 2). The photocytotoxic efficacy of the studied compound is shown in Figure 3A. At a dose of 2.5 µM, Glu-4-ZnPc-P emerged as the most potent cytotoxic compound in our study, underscoring its potential efficacy in inducing cellular cytotoxicity. Interestingly, after 24 h of dark incubation without irradiation, the cytotoxic activity of the ZnPc–sugar conjugates was lower at higher concentrations compared to ZnPc (Figure 3B). Our findings align with several studies conducted on breast cancer cells [27,28], demonstrating that acetyl-protected conjugates exhibiting superior photodynamic activity exhibit lower intracellular uptake, thereby implying that uptake alone is not the predominant factor influencing photodynamic damage. One potential explanation could involve the intracellular localization of the compound, where it may accumulate in non-cytotoxic compartments or fail to effectively interact with critical cellular targets. Additionally, the compound’s chemical properties or structural characteristics might hinder its ability to induce cytotoxic effects despite efficient cellular internalization. Moreover, the administered light dose in our study, at 0.9 J/cm^2^, was comparatively smaller than those previously utilized for the activation of other metal-based photocytotoxic compounds in HaCaT cells, such as the α-(8-Quinolinoxy) zinc phthalocyanine (ZnPc-F7), with a fluence of 5.37 J/cm^2^ [20].

**Figure 4:**
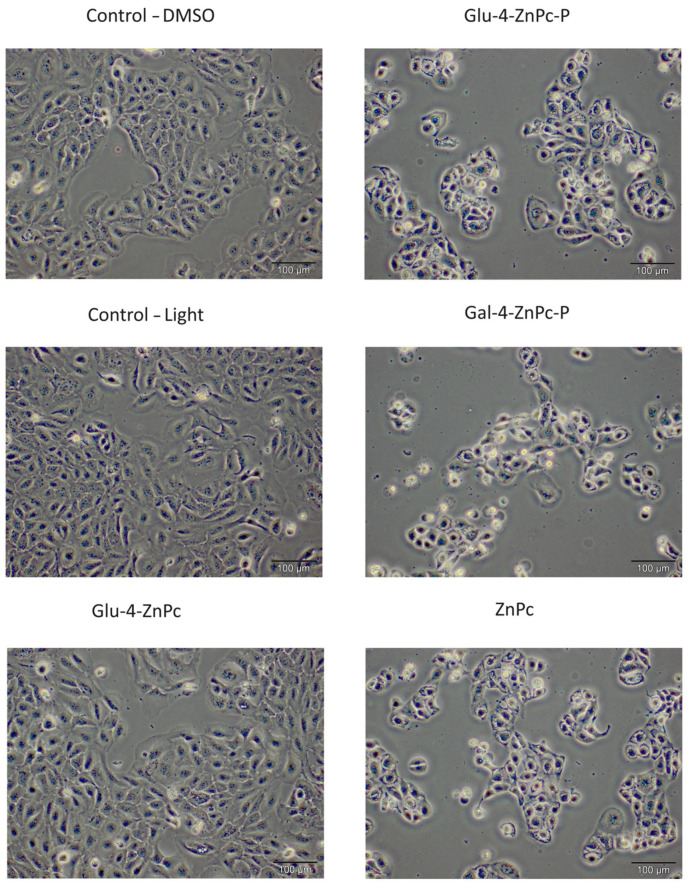
The morphology of cytokine-stimulated HaCaT cells 24 h after photodynamic effect with sugar-conjugated ZnPc and free ZnPc at a drug dose of 2.5 µM and light dose of 0.9 J/cm^2^.

To better illustrate the phototoxicity of the studied photosensitizers, we also examined the morphological changes in HaCaT cells after PDT using optical microscopy (Figure 4). The presented images revealed that the treated cells were partially or completely dead 24 h after photodynamic treatment. The residual cells primarily manifested characteristics such as cellular shrinkage, detachment, diminished refractivity, and morphological changes indicative of cell death processes, including apoptosis and necrosis. In summary, these findings provide evidence of the cytotoxic impact exerted by the conjugates on HaCaT cells stimulated with IL-6, IL-17A, and IL-23.

### 3.2. Proinflammatory Cytokines IL-6, IL-17A, and IL-23 Enhance the Cellular Uptake of Glu-4-ZnPc and Glu-4-ZnPc-P, While the Selective GLUT1 Inhibitor BAY-876 Reduces the Accumulation of the Compounds in HaCaT Cells

**Figure 5:**
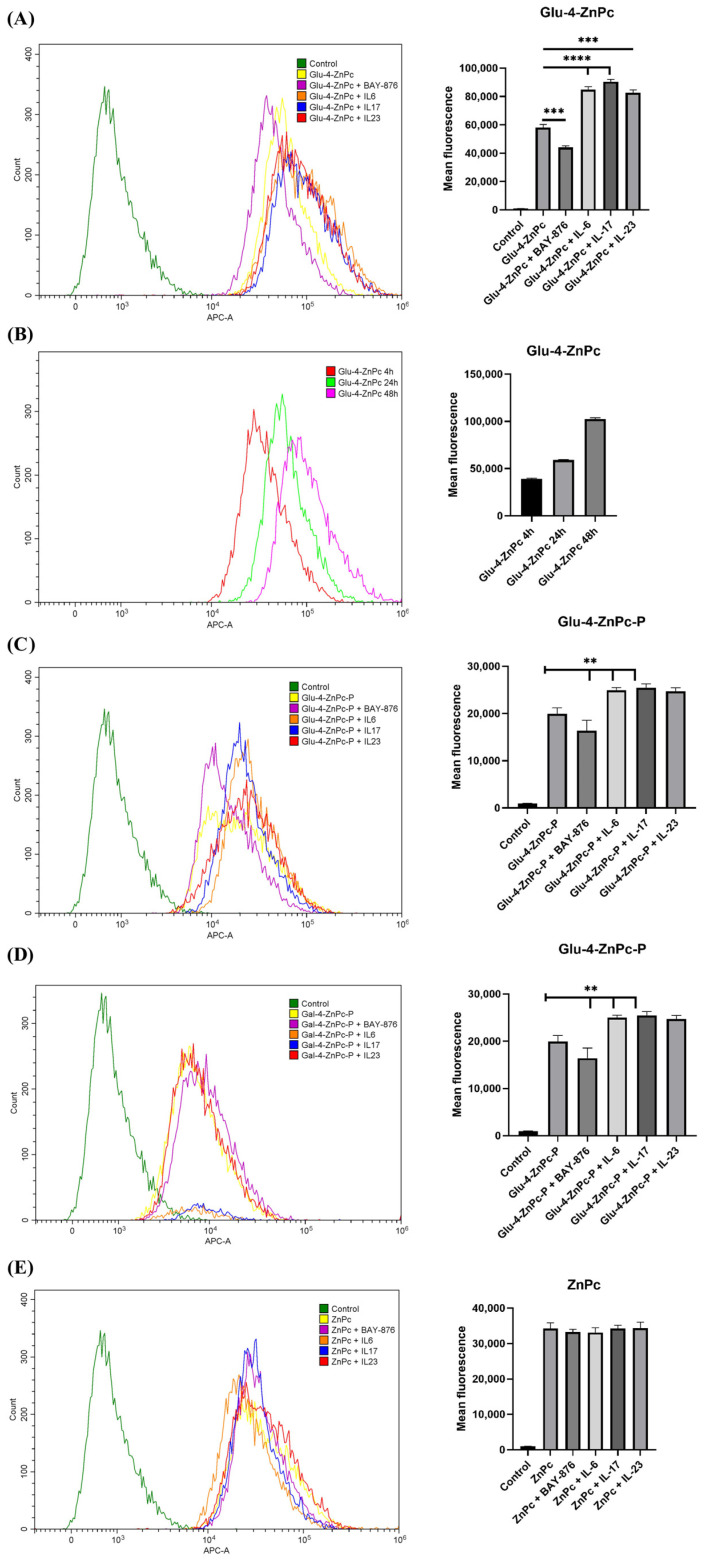
Flow cytometry analysis of uptake of sugar-conjugated zinc phthalocyanines compared to free ZnPc, after stimulation with proinflammatory cytokines IL-6, IL-17A, and IL-23 or inhibition with BAY-876; (**A**) Glu-4-ZnPc after 24 h; (**B**) Glu-4-ZnPc after 4 h, 24 h and 48 h; (**C**) Glu-4-ZnPc-P after 24 h; (**D**) Gal-4-ZnPc-P after 24 h; (**E**) ZnPc after 24 h. ** (*p* < 0.01); *** (*p* < 0.001); **** (*p* < 0.0001).

The cellular uptake of Glu-4-ZnPc was significantly influenced by the presence of proinflammatory cytokines IL-6, IL-17A, and IL-23. Specifically, IL-17A demonstrated the most substantial enhancement, with a mean fluorescence intensity increase of 56.3%. IL-6 and IL-23 also increased the cellular uptake by 46.6% and 42.2%, respectively (Figure 5A). The selective GLUT1 inhibitor BAY-876 exhibited a notable reduction in the accumulation of Glu-4-ZnPc. The mean fluorescence intensity was decreased by 23.8% for the cells treated with the inhibitor before treatment. Additionally, the results show a time-dependent progressive increase in Glu-4-ZnPc uptake over a 48 h period (Figure 5B).

In addition to Glu-4-ZnPc, we investigated the cellular uptake of the protected glucose–ZnPc conjugate Glu-4-ZnPc-P under the same conditions. After treatment with IL-6, the uptake increased significantly by 25.4% from 19,919 to 24,961 mean fluorescence. Similarly, IL-17A and IL-23 treatments resulted in elevated uptake, with percentage differences of 27.6% and 23.9%, respectively (*p* < 0.01 for all cytokines) (Figure 5C).

BAY-876 treatment led to a reduction in Glu-4-ZnPc-P uptake, with a mean fluorescence intensity decrease of 17.9%. However, this reduction was not statistically significant (*p* = 0.072).

Unlike Glu-4-ZnPc and Glu-4-ZnPc-P, the mean fluorescence intensity of HaCaT cells treated with Gal-4-ZnPc-P or ZnPc did not increase upon stimulation with proinflammatory cytokines IL-6, IL-17A, and IL-23, nor decrease following incubation with BAY-876 (Figure 5D,E). In summary, the mean fluorescence intensity analysis reveals a distinct hierarchy in cellular uptake among the studied compounds: Glu-4-ZnPc > ZnPc > Glu-4-ZnPc-P > Gal-4-ZnPc-P.

In our previous study, we showed that the incubation of HaCaT cells with proinflammatory cytokines, such as IL-6, IL-17A, IL-23, and IL-36, enhances the expression of GLUT1 transporter [29]. Here, for the first time, we noticed that sugar-conjugated zinc phthalocyanines are preferentially accumulated in the intracellular compartment of the hyperproliferating keratinocytes and that the unprotected Glu-4-ZnPc is partially transported by GLUT1 inhibitor. In the pursuit of prodrugs selectively targeted to treated cells, emphasis has been placed on constructs containing hexoses, including D-glucose, D-galactose, D-mannose, and D-fructose. Utilizing the overexpression of GLUT1 in hyperproliferating cells, as opposed to normal cells, has emerged as an appealing strategy for the controlled delivery of prodrugs formed by conjugating biologically active aglycons with sugar derivatives [30].

### 3.3. Sugar–Zinc Phthalocyanine Conjugates Are Preferentially Accumulated in Mitochondria

**Figure 6:**
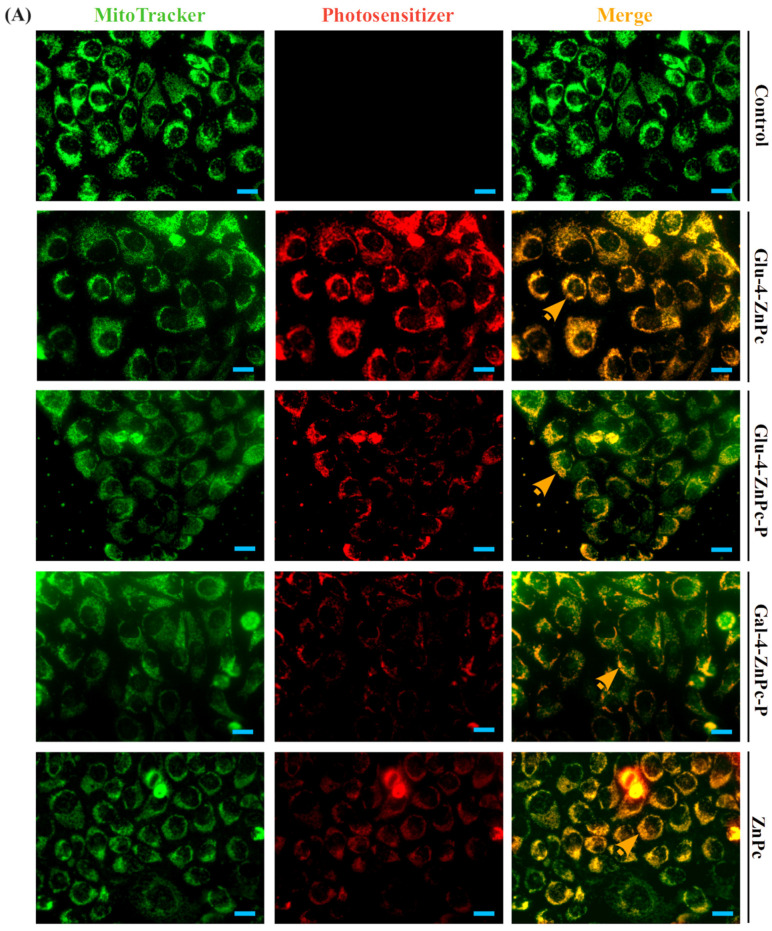

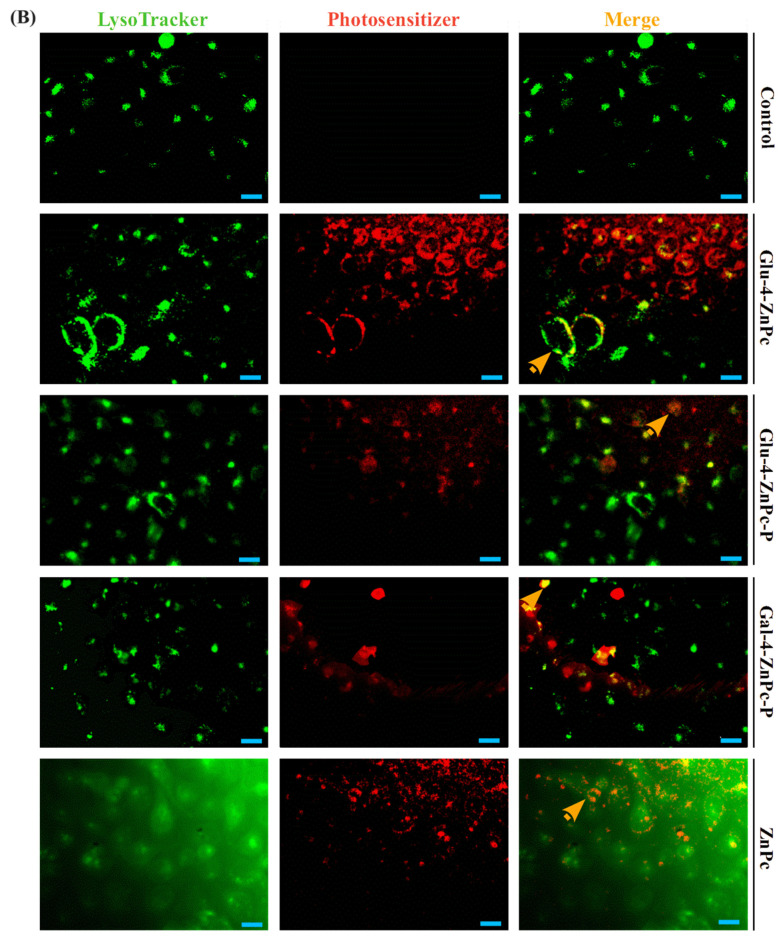

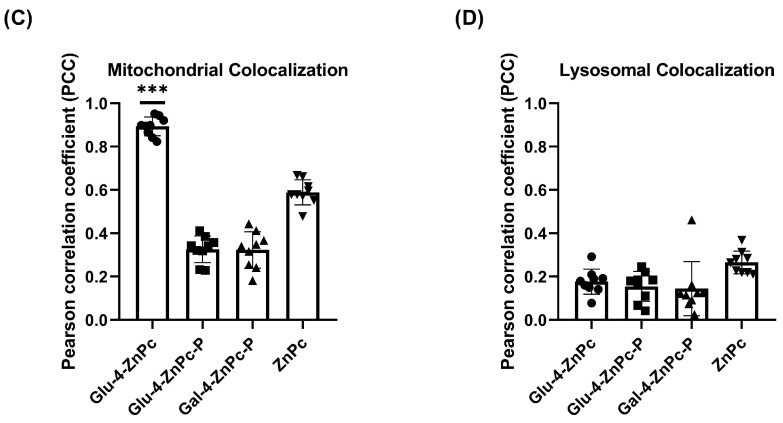
Subcellular localization of sugar-conjugated zinc phthalocyanine and free ZnPc in HaCaT cells after 24 h incubation. The red fluorescence of the compounds was mainly colocalized with (**A**) MitoTracker^®^. In contrast, the red fluorescence of the compounds displayed scant colocalization with (**B**) LysoTracker^®^ green fluorescence; Pearson’s correlation coefficients (PCCs) of images (*n* = 9) of phthalocyanines and green MitoTracker^®^ (**C**,**D**) LysoTracker^®^ in HaCaT cells using Pearson’s correlation coefficient and one-way ANOVA. *n* = 9 images for each group. *** *p* < 0.001. Orange arrows show areas of colocalization of autofluorescent signal with mitochondrial or lysosomal probes. Scale bars = 20 μm.

To confirm the targeted specificity of sugar-conjugated zinc phthalocyanines toward HaCaT cells, a medium containing 2.5 μM of each compound was administrated for 24 h. The fluorescence images showed that all of the examined drugs were predominantly localized in mitochondria, with a clear colocalization with MitoTracker^®^, contrary to the lysosome marker LysoTracker^®^ (Figure 6). Glu-4-ZnPc showed a significantly higher colocalization with mitochondria compared to other phthalocyanines (*p* < 0.001) (Figure 6C). Acetyl-protected Glu-4-ZnPc-P and Gal-4-ZnPc-P displayed a lower intracellular accumulation compared to unprotected Glu-4-ZnPc and free ZnPc. The results are in line with those obtained by flow cytometry. These findings prove the increased intracellular accumulation of unprotected glucose–zinc phthalocyanine in HaCaT cells. In contrast to previous findings suggesting inadequate localization of phthalocyanines within mitochondria due to a dispersed distribution [27], our study demonstrates successful accumulation of glucosylated zinc phthalocyanines within the mitochondria. Despite differences in cytotoxic mechanisms and the observed effects of the investigated sugar-conjugated phthalocyanines, all of the studied compounds exhibited a preference for subcellular localization in the mitochondria. These organelles have been extensively studied as focal points for optimizing the efficacy of PDT due to their vital roles in cellular metabolism [31,32,33]. Targeted PDT for mitochondria is achieved using phthalocyanine photosensitizers facilitated by carrier delivery and structural modification involving aromatic nitrogen heterocycles, ammonium, TPP, or polypeptides [34]. However, despite certain significant findings [35], the association between mitochondria and psoriasis has remained unclear until now.

### 3.4. Glu-4-ZnPc-P Photogenerates Intracellular ROS the Most Efficiently in HaCaT Cells

**Figure 7:**
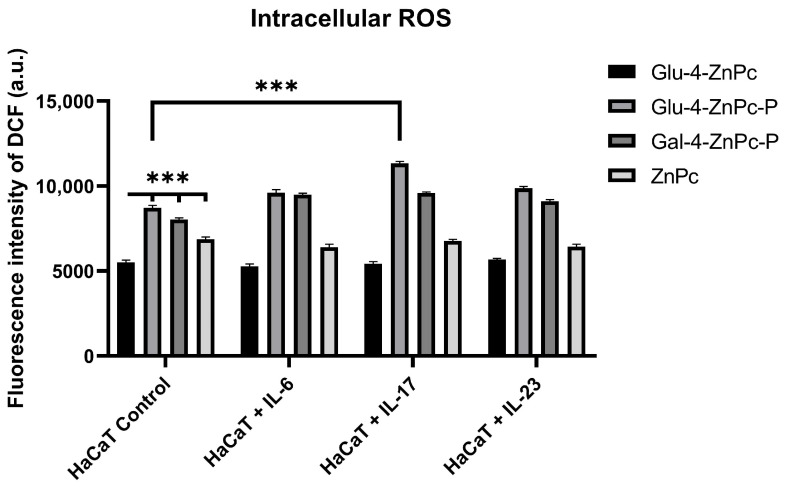
Cellular ROS generation efficiency Glu-4-ZnPc, Glu-4-ZnPc-P, Gal-4-ZnPc-P, and ZnPc (all at 2.5 µM) with the light dose of 0.9 J/cm^2^; *** (*p* < 0.001).

The irradiation of the sugar-conjugated zinc phthalocyanines led to the production of ROS, one of the main mediators of cellular death induced by PDT. The intracellular ROS generation efficiency of the studied compounds against control HaCaT cells or stimulated with exogenous cytokines was examined by using DCF, an oxidized green fluorescent product of 2′,7′-dichlorodihydrofluorescein diacetate (DCFH-DA). We attempted to compare the formation of ROS between different phthalocyanines. The results show that all of the analyzed phthalocyanines can induce the formation of ROS under illumination, with the efficiency at generating ROS in the following order: Glu-4-ZnPc-P > Gal-4-ZnPc-P > ZnPc >> Glu-4-ZnPc (*p* < 0.001, Figure 7), which is strongly correlates the with in vitro photocytotoxicity. Moreover, we observed that the preincubation of HaCaT cells with IL-17A significantly increases the intracellular ROS generation following Glu-4-ZnPc-P-PDT treatment (*p* < 0.001). Our study aligns with the results obtained by Liu et al. [27] and Kimani et al. [28], who showed that acetyl-protected conjugates generate higher ROS levels compared to unprotected sugar-conjugated zinc phthalocyanines on MCF-7 breast cancer cells. One possible explanation for these results could be the presence of subcellular localization or compartmentalization of the photosensitizer within specific cellular organelles, leading to localized ROS production even in the context of overall low cellular uptake. Additionally, the efficiency of ROS generation might be influenced by the photosensitizer’s interaction with intracellular targets. It is worth noting that apart from IL-17, which increased the intracellular ROS generation following Glu-4-ZnPc-P-PDT treatment, the presence of proinflammatory cytokines did not affect the intracellular ROS generation. This could be attributed to the inherent photostability and quantum yield of Glu-4-ZnPc, which remained unaffected by cytokines. Additionally, factors such as enhanced antioxidant defenses or cellular stress responses might mitigate ROS production, leading to low cell cytotoxicity despite the higher intracellular concentration of Glu-4-ZnPc. Further investigations into the subcellular distribution, intracellular trafficking, and molecular interactions of the photosensitizer may provide insights into the mechanisms underlying high ROS levels despite low cellular uptake, contributing to a comprehensive understanding of its therapeutic potential.

### 3.5. In Vivo Antipsoriatic Activity of Glu-4-ZnPc-P

**Figure 8:**
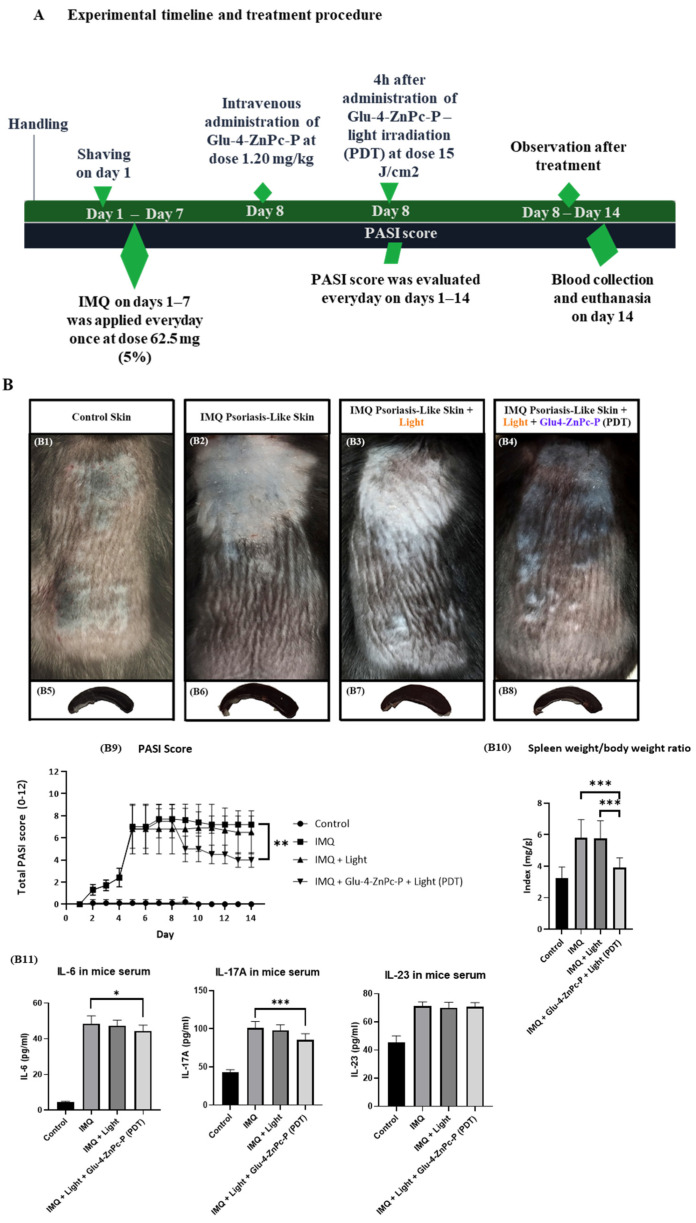

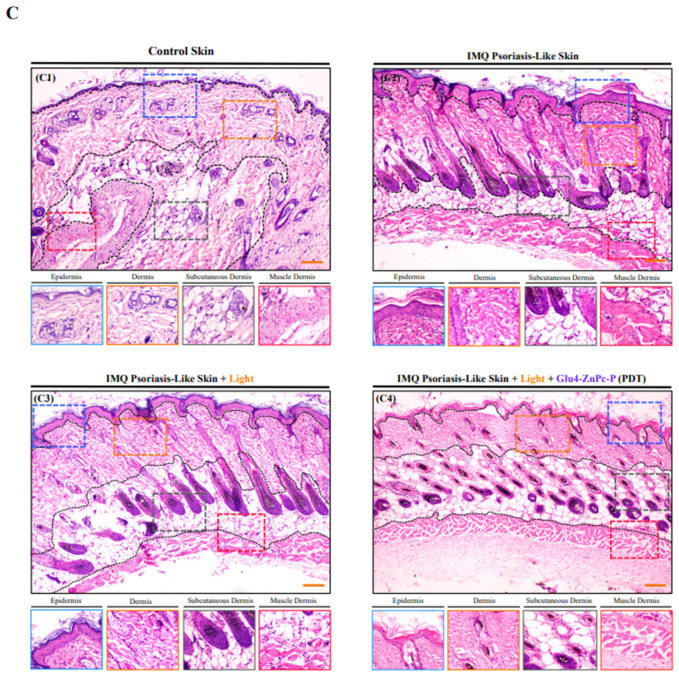
(**A**) The diagram illustrates the steps of experimental design in time. (**B**) The safety and therapeutic effects of PDT were analyzed in several ways. The daily imaging of skin backs of (**B1**): control skin; (**B2**): IMQ-Psoriasis-Like Skin; (**B3**): IMQ-Psoriasis-Like Skin with Light irradiation; (**B4**): complete PDT: IMQ-Psoriasis-Like Skin following Light Irradiation and intravenous administration of Glu-4-ZnPc-P; (**B5**–**B8**): macroscopic assessment of spleens in the studied groups; (**B9**): Total PASI score; (**B10**): calculation of spleen/body weight ratio index; (**B11**): determination of IL-6, IL-17A and IL-23 levels in mice serum by ELISA; (**C**) hematoxylin and eosin (H&E) staining of all skin compartments, including the epidermis, dermis, subcutaneous dermis and muscle dermis in (**C1**): control group; (**C2**): IMQ-Psoriatic-like skin; (**C3**) IMQ-Psoriasis-Like Skin with Light irradiation; (**C4**): Glu-4-ZnPc-P-PDT. The whole skin tissue from all four groups was additionally selected as squares and enlarged below main images for epidermis (blue), dermis (orange), subcutaneous (gray), and muscle (red) dermis for better visualization of skin compartments. * (*p* < 0.05); ** (*p* < 0.01); *** (*p* < 0.001); the main images are under magnification of ×10, scale bars = 100 µm.

In light of the more promising in vitro results observed with Glu-4-ZnPc-P, we attempted to verify its safety and therapeutic efficacy in vivo. The skin of mice from each group was monitored daily: Control skin (B1), IMQ-Psoriasis-Like Skin (B2), IMQ-Psoriasis-Like Skin with Red Light irradiation (B3), and PDT: IMQ-Psoriasis-Like Skin following intravenous administration of Glu-4-ZnPc-P and red light irradiation (B4) (Figure 8). The PASI index was calculated based on the daily monitoring of skin condition. The PDT-treated group demonstrates significant improvement in PASI index compared to the skin of the IMQ-Psoriasis-Like group (*p* < 0.0052) (Figure 8(B9)). Moreover, the impact of PDT was analyzed by macroscopic assessment of spleens in all groups, as well by calculation of spleen/body weight ratio index (Figure 8(B5–B8,B10)). Spleens of animals after Glu-4-ZnPc-P-PDT have significantly inhibited IMQ-related splenomegaly in relation to IMQ-Psoriasis-Like Skin (*p* < 0.001) and Light Irradiated IMQ-Psoriasis-Like Skin group, respectively (*p* < 0.001). Additionally, we evaluated the impact of Glu-4-ZnPc-P-PDT therapy on the level of proinflammatory cytokines in mice serum. We found that IL-6 (*p* < 0.05) and IL-17A (*p* < 0.001) were significantly lower 7 days after irradiation (Figure 8(B11)). Finally, hematoxylin and eosin (H&E) staining and assessment by experienced histopathologists confirm the effectiveness of Glu-4-ZnPc-P-PDT (Figure 8(C1–C4)). The analysis confirmed the restoring normal morphology, skin barrier and thickness, as well as the visible reduction in leukocyte infiltration in all skin compartments, including the epidermis, dermis, subcutaneous dermis, and muscle dermis in PDT animals (C4). The skin of the PDT group demonstrates comparable morphological and cellular parameters to the skin of the control group (C1) but opposite to incomplete only light irradiated (C3) and IMQ-Psoriatic-like skin (C2) groups of animals, respectively. Our study shows the promising realm of PDT in psoriasis treatment in comparison with previous investigations that have explored zinc phthalocyanines as potential photosensitizers. A study conducted by Liu et al. [20] revealed the anti-psoriasis effects of α-(8-quinolinoxy) zinc phthalocyanine (ZnPc-F7)-mediated PDT, showcasing its inhibitory impact on hyperproliferation and immune regulation in both in vitro and in vivo models. This aligns with our findings, where Glu-4-ZnPc-P exhibited significant inhibitory effects on HaCaT cell proliferation and attenuated the psoriasis-like inflammation in mice.

## 4. Conclusions

In conclusion, our study underscores the potential of sugar-conjugated zinc phthalocyanines, particularly tetrasubstituted protected glucose–ZnPc (Glu-4-ZnPc-P), as promising antipsoriatic agents. Demonstrating superior phototoxicity and enhanced cellular uptake in cytokine-stimulated keratinocytes, Glu-4-ZnPc-P proved effective in inhibiting abnormal keratinocyte proliferation. Moreover, the in vivo application of Glu-4-ZnPc-P photodynamic therapy demonstrated significant improvement in Psoriasis Area and Severity Index (PASI), effectively inhibiting splenomegaly and restoring normal skin morphology in an established IMQ psoriasis-like model. Despite a few promising outcomes, the clinical utilization of PDT in psoriasis treatment is still limited. This emphasizes the importance of conducting additional research to assess the effectiveness of a broader range of photosensitizers and explore the potential of targeted photodynamic therapy in the treatment of psoriasis.

## Data Availability

Data are contained within the article.

## Supplementary Materials

The following supporting information can be downloaded at https://www.mdpi.com/article/10.3390/pharmaceutics16060838/s1, Figure S1: FT-IR spectrum of Glu-4-ZnPc in KBr; Figure S2: ^1^H NMR (600.15 MHz) of Glu-4-ZnPc in DMSO-*d*_6_; Figure S3: ^1^H NMR (600.15 MHz) of Glu-4-ZnPc in DMF-*d*_7_; Figure S4: MALDI-TOF of Glu-4-ZnPc. Inset: Experimental isotopic distribution pattern for Glu-4-ZnPc; Figure S5: Simulated isotopic distribution pattern for Glu-4-ZnPc; Figure S6: HPLC analysis of Glu-4-ZnPc using 280 nm detection; Figure S7: HPLC analysis of Glu-4-ZnPc using 670 nm detection; Figure S8: Emission spectra of Glu-4-ZnPc in H_2_O and DMSO; Figure S9: Excitation spectra of Glu-4-ZnPc in H_2_O and DMSO.

## References

[1] Parisi R., Iskandar I.Y.K., Kontopantelis E., Augustin M., Griffiths C.E.M., Ashcroft D.M. (2020). National, Regional, and Worldwide Epidemiology of Psoriasis: Systematic Analysis and Modelling Study. BMJ.

[2] Armstrong A.W., Read C. (2020). Pathophysiology, Clinical Presentation, and Treatment of Psoriasis: A Review. JAMA—J. Am. Med. Assoc..

[3] Lowes M.A., Bowcock A.M., Krueger J.G. (2007). Pathogenesis and Therapy of Psoriasis. Nature.

[4] Rendon A., Schäkel K. (2019). Psoriasis Pathogenesis and Treatment. Int. J. Mol. Sci..

[5] Yamanaka K., Yamamoto O., Honda T. (2021). Pathophysiology of Psoriasis: A Review. J. Dermatol..

[6] Menter A., Gelfand J.M., Connor C., Armstrong A.W., Cordoro K.M., Davis D.M.R., Elewski B.E., Gordon K.B., Gottlieb A.B., Kaplan D.H. (2020). Joint American Academy of Dermatology–National Psoriasis Foundation Guidelines of Care for the Management of Psoriasis with Systemic Nonbiologic Therapies. J. Am. Acad. Dermatol..

[7] Kwiatkowski S., Knap B., Przystupski D., Saczko J., Kędzierska E., Knap-Czop K., Kotlińska J., Michel O., Kotowski K., Kulbacka J. (2018). Photodynamic Therapy—Mechanisms, Photosensitizers and Combinations. Biomed. Pharmacother..

[8] Allison R.R., Moghissi K. (2013). Photodynamic Therapy (PDT): PDT Mechanisms. Clin. Endosc..

[9] Gunaydin G., Gedik M.E., Ayan S. (2021). Photodynamic Therapy for the Treatment and Diagnosis of Cancer—A Review of the Current Clinical Status. Front. Chem..

[10] Choi Y.M., Adelzadeh L., Wu J.J. (2015). Photodynamic Therapy for Psoriasis. J. Dermatol. Treat..

[11] Wöhrle D., Schnurpfeil G., Makarov S.G., Kazarin A., Suvorova O.N. (2012). Practical Applications of Phthalocyanines—From Dyes and Pigments to Materials for Optical, Electronic and Photo-Electronic Devices. Macroheterocycles.

[12] Kadish K.M., Smith K.M., Guilard R. (2000). The Porphyrin Handbook.

[13] Durmuş M., Nyokong T. (2007). Synthesis, Photophysical and Photochemical Studies of New Water-Soluble Indium(III) Phthalocyanines. Photochem. Photobiol. Sci..

[14] Roguin L.P., Chiarante N., García Vior M.C., Marino J. (2019). Zinc(II) Phthalocyanines as Photosensitizers for Antitumor Photodynamic Therapy. Int. J. Biochem. Cell Biol..

[15] Jin Y., Zhang X., Zhang B., Kang H., Du L., Li M. (2015). Nanostructures of an Amphiphilic Zinc Phthalocyanine Polymer Conjugate for Photodynamic Therapy of Psoriasis. Colloids Surf. B Biointerfaces.

[16] Bächle F., Siemens N., Ziegler T. (2019). Glycoconjugated Phthalocyanines as Photosensitizers for PDT—Overcoming Aggregation in Solution. Eur. J. Org. Chem..

[17] Castro K.A.D.F., Prandini J.A., Biazzotto J.C., Tomé J.P.C., da Silva R.S., Lourenço L.M.O. (2022). The Surprisingly Positive Effect of Zinc-Phthalocyanines With High Photodynamic Therapy Efficacy of Melanoma Cancer. Front. Chem..

[18] Kuzyniak W., Schmidt J., Glac W., Berkholz J., Steinemann G., Hoffmann B., Ermilov E.A., Görek A.G., Ahsen V., Nitzsche B. (2017). Novel Zinc Phthalocyanine as a Promising Photosensitizer for Photodynamic Treatment of Esophageal Cancer. Int. J. Oncol..

[19] Makuch S., Dróżdż M., Makarec A., Ziółkowski P., Woźniak M. (2022). An Update on Photodynamic Therapy of Psoriasis—Current Strategies and Nanotechnology as a Future Perspective. Int. J. Mol. Sci..

[20] Liu H.Q., Wang Y.M., Li W.F., Li C., Jiang Z.H., Bao J., Wei J.F., Jin H.T., Wang A.P. (2017). Anti-Psoriasis Effects and Mechanisms of A-(8-Quinolinoxy) Zinc Phthalocyanine-Mediated Photodynamic Therapy. Cell. Physiol. Biochem..

[21] Zhang Z., Zi Z., Lee E.E., Zhao J., Contreras D.C., South A.P., Abel E.D., Chong B.F., Vandergriff T., Hosler G.A. (2018). Differential Glucose Requirement in Skin Homeostasis and Injury Identifies a Therapeutic Target for Psoriasis Article. Nat. Med..

[22] Hodeib A.A.H., Neinaa Y.M.E.H., Zakaria S.S., Alshenawy H.A.S. (2018). Glucose Transporter-1 (GLUT-1) Expression in Psoriasis: Correlation with Disease Severity. Int. J. Dermatol..

[23] Choi C.-F., Huang J.-D., Lo P.-C., Fong W.-P., Ng D.K.P. (2008). Glycosylated Zinc(Ii) Phthalocyanines as Efficient Photosensitisers for Photodynamic Therapy. Synthesis, Photophysical Properties and in Vitro Photodynamic Activity. Org. Biomol. Chem..

[24] Ribeiro A.O., Tomé J.P.C., Neves M.G.P.M.S., Tomé A.C., Cavaleiro J.A.S., Iamamoto Y., Torres T. (2006). [1,2,3,4-Tetrakis(α/β-d-Galactopyranos-6-Yl)Phthalocyaninato]Zinc(II): A Water-Soluble Phthalocyanine. Tetrahedron Lett..

[25] Dunn K.W., Kamocka M.M., McDonald J.H. (2011). A Practical Guide to Evaluating Colocalization in Biological Microscopy. Am. J. Physiol. Cell Physiol..

[26] Wang M., Ishii K. (2022). Photochemical Properties of Phthalocyanines with Transition Metal Ions. Coord. Chem. Rev..

[27] Liu J.Y., Wang C., Zhu C.H., Zhang Z.H., Xue J.P. (2017). Preparation and In Vitro Photodynamic Activity of Glucosylated Zinc(II) Phthalocyanines as Underlying Targeting Photosensitizers. Molecules.

[28] Kimani S.G., Shmigol T.A., Hammond S., Phillips J.B., Bruce J.I., MacRobert A.J., Malakhov M.V., Golding J.P. (2013). Fully Protected Glycosylated Zinc (II) Phthalocyanine Shows High Uptake and Photodynamic Cytotoxicity in MCF-7 Cancer Cells. Photochem. Photobiol..

[29] Makuch S., Kupczyk P., Makarec A., Chodaczek G., Ziółkowski P., Woźniak M. (2023). The Impact of Proinflammatory Cytokines and Imiquimod on GLUT1 in HaCaT Keratinocytes—A Potential Anti-Psoriatic Therapeutic Target?. Cell. Physiol. Biochem..

[30] Makuch S., Wozniak M., Krawczyk M., Pastuch-Gawołek G., Szeja W., Agrawal S. (2020). Glycoconjugation as a Promising Treatment Strategy for Psoriasis. J. Pharmacol. Exp. Ther..

[31] Ge Y., Weng X., Tian T., Ding F., Huang R., Yuan L., Wu J., Wang T., Guo P., Zhou X. (2013). A Mitochondria-Targeted Zinc(II) Phthalocyanine for Photodynamic Therapy. RSC Adv..

[32] Muli D.K., Rajaputra P., You Y., McGrath D.V. (2014). Asymmetric ZnPc-Rhodamine B Conjugates for Mitochondrial Targeted Photodynamic Therapy. Bioorg. Med. Chem. Lett..

[33] Nash G.T., Luo T., Lan G., Ni K., Kaufmann M., Lin W. (2021). Nanoscale Metal-Organic Layer Isolates Phthalocyanines for Efficient Mitochondria-Targeted Photodynamic Therapy. J. Am. Chem. Soc..

[34] Li M., Zheng K., Liu X. (2023). Mitochondria-Targeting Phthalocyanines and Porphyrins for Enhanced Photodynamic Tumor Therapy. ChemistrySelect.

[35] Mizuguchi S., Gotoh K., Nakashima Y., Setoyama D., Takata Y., Ohga S., Kang D. (2021). Mitochondrial Reactive Oxygen Species Are Essential for the Development of Psoriatic Inflammation. Front. Immunol..

