# Supplementary material for: In Vitro and In Vivo Antipsoriatic Efficacy of Protected and Unprotected Sugar–Zinc Phthalocyanine Conjugates
Source: Pharmaceutics. 2024 Jun 20;16(6):838. doi: 10.3390/pharmaceutics16060838 (PMC11207564; doi:10.3390/pharmaceutics16060838)
Supplement: Supplementary file 1 [file pharmaceutics-16-00838-s001.zip › pharmaceutics-3013719-supplementary.pdf]

Supplementary material

Table of Contents

Supplementary Figures

Figure S1. FT-IR spectrum of Glu-4-ZnPc in KBr.
Figure S2. ^{1}H NMR (600.15 MHz) of Glu-4-ZnPc in DMSO-d_6.
Figure S3. ^{1}H NMR (600.15 MHz) of Glu-4-ZnPc in DMF-d_7.
Figure S4. MALDI-TOF of Glu-4-ZnPc. Inset: Experimental isotopic distribution pattern for Glu-4-ZnPc.
Figure S5. Simulated isotopic distribution pattern for Glu-4-ZnPc.
Figure S6. HPLC analysis of Glu-4-ZnPc using 280 nm detection.
Figure S7. HPLC analysis of Glu-4-ZnPc using 670 nm detection.
Figure S8. Emission spectra of Glu-4-ZnPc in H_2O and DMSO.
Figure S9. Excitation spectra of Glu-4-ZnPc in H2O and DMSO.

Supplementary Figures

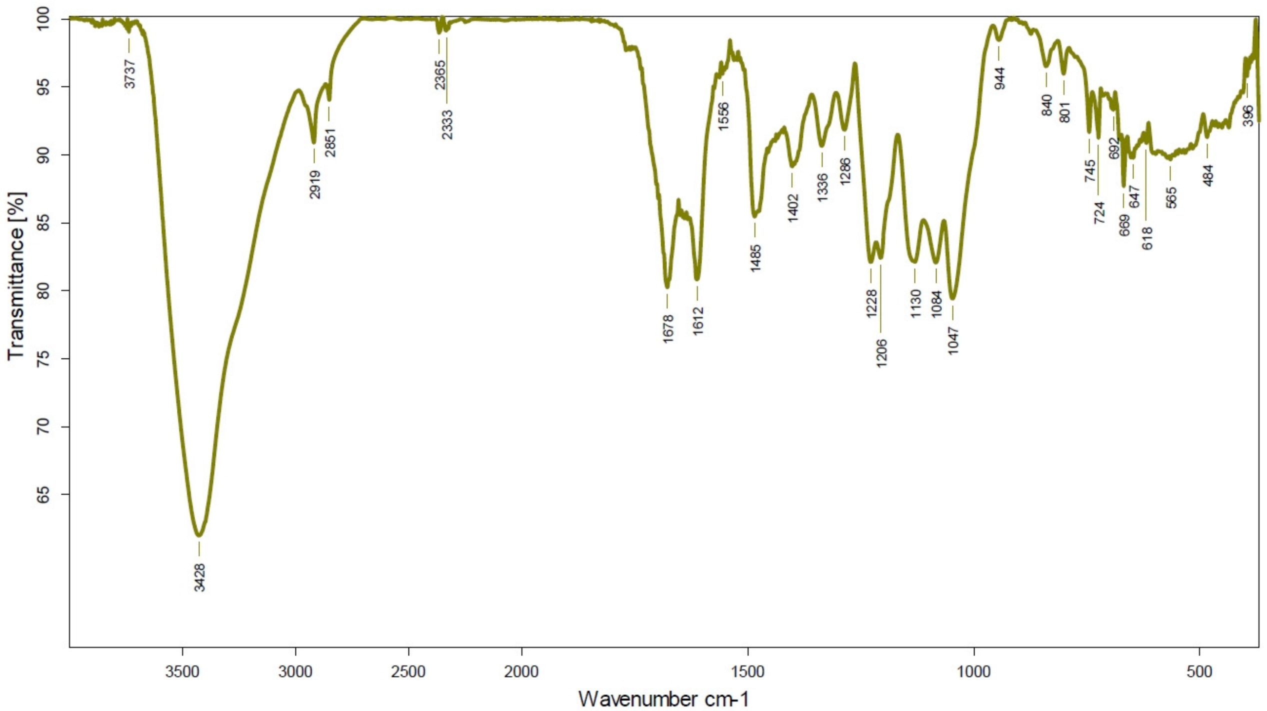

Figure S1. FT-IR spectrum of Glu-4-ZnPc in KBr.

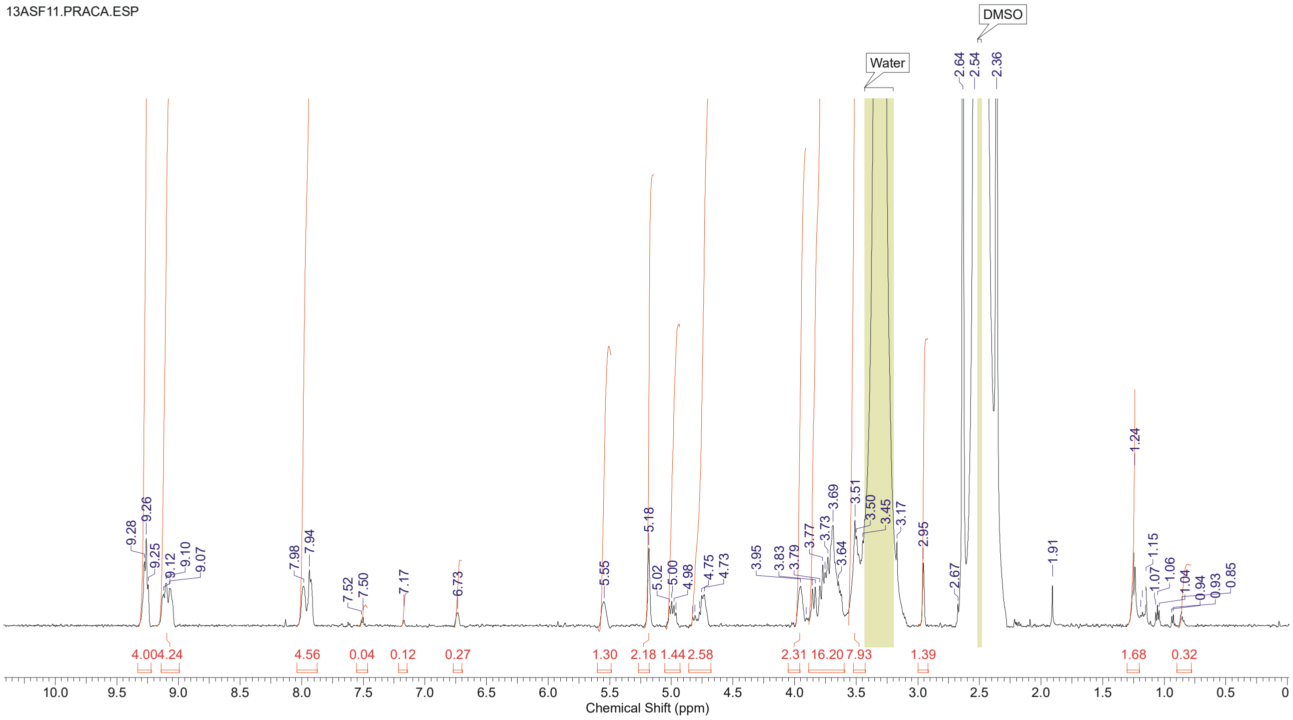

Figure S2. ^{1}H NMR (600.15 MHz) of Glu-4-ZnPc in DMSO-d_6.

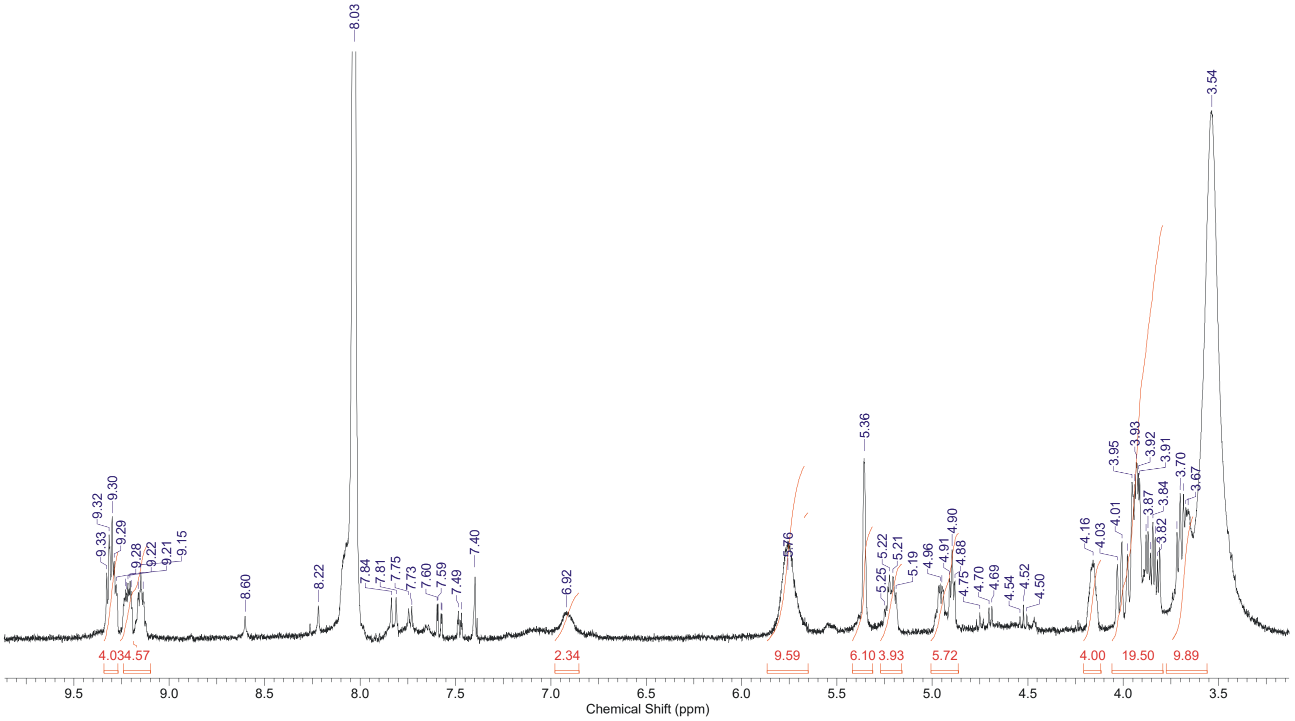

Figure S3. ^{1}H NMR (600.15 MHz) of Glu-4-ZnPc in DMF-d_7 with trace amount of pirydine-d_5.

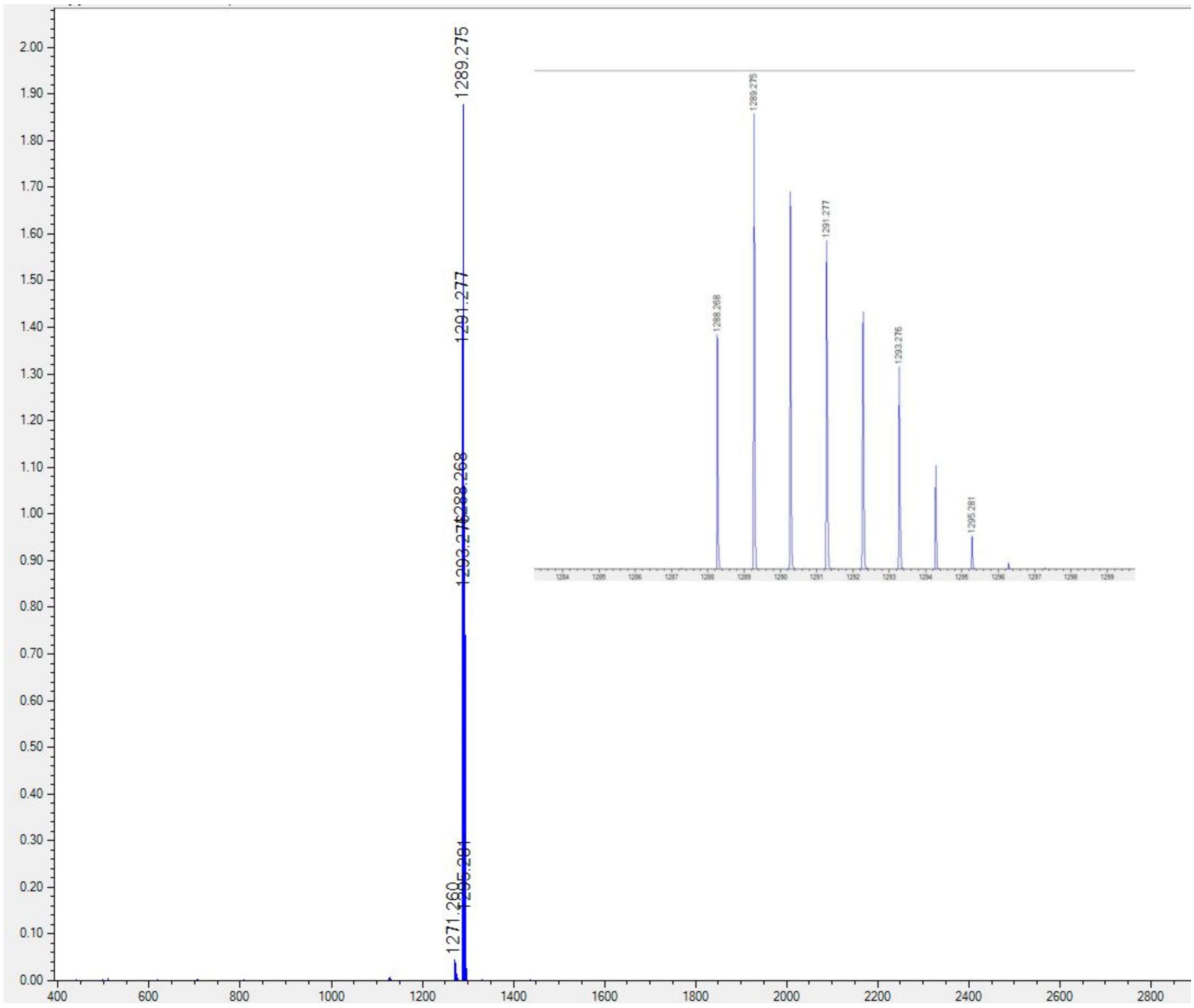

Figure S4. MALDI-TOF of Glu-4-ZnPc. Inset: Experimental isotopic distribution pattern for 1.

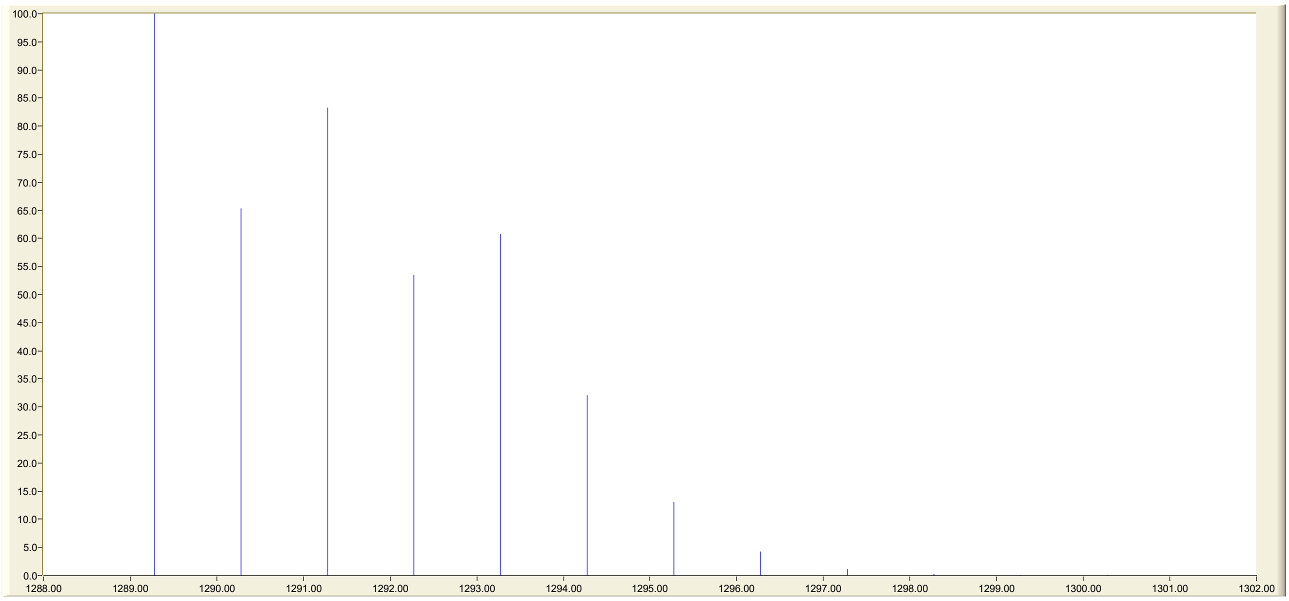

Figure S5. Simulated isotopic distribution pattern for Glu-4-ZnPc.

Operator:W³aœciciel Timebase:Dionex_LC Sequence:kolejka_1 Page 1-1
2024-4-8 4:44 PM

4 20221220_AKL_PS_G10_fr3

Sample Name:	**20221220_AKL_PS_G10_fr3**	*Injection Volume:*	**30,0**
Vial Number:	**3**	*Channel:*	**UV_VIS_2**
Sample Type:	**unknown**	*Wavelength:*	**280**
Control Program:	**0_80_30_VIS_akl**	*Bandwidth:*	**n.a.**
Quantif. Method:	**integracja_PS**	*Dilution Factor:*	**1,0000**
Recording Time:	**2022-12-20 16:18**	*Sample Weight:*	**1,0000**
Run Time (min):	**37,00**	*Sample Amount:*	**1,0000**

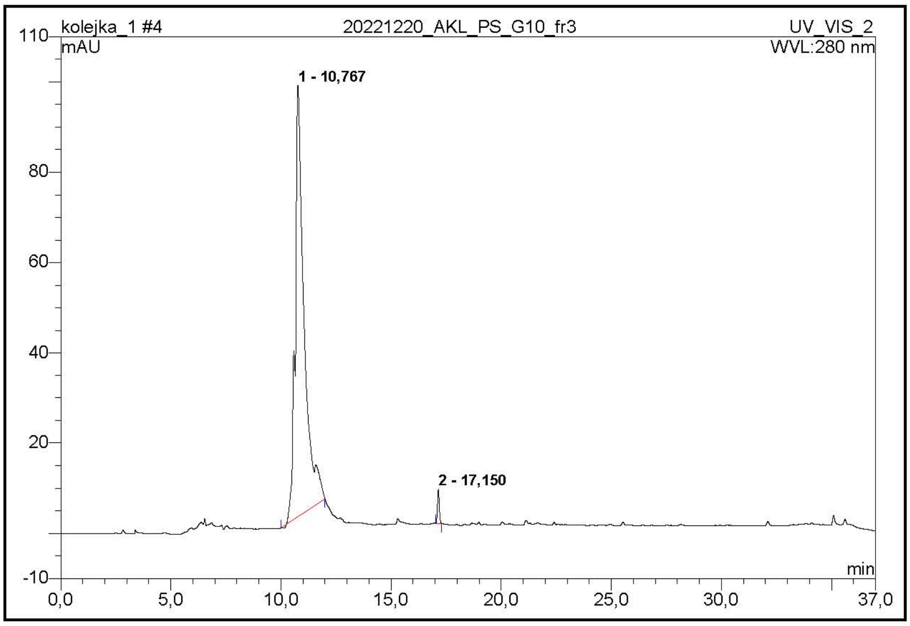

No.	Ret.Time min	Peak Name	Height mAU	Area mAU*min	Rel.Area %	Amount	Type
1	10,77	n.a.	95,592	44,447	98,41	n.a.	BMB
2	17,15	n.a.	7,554	0,718	1,59	n.a.	BMB
**Total:**			103,146	45,165	100,00	0,000	

DEFAULT/Integration
Chromeleon (c) Dionex 1996-2006
Version 6.80 SR6 Build 2491 (141932)

Figure S6. HPLC analysis of Glu-4-ZnPc using 280 nm detection.

4 20221220_AKL_PS_G10_fr3

Sample Name:	**20221220_AKL_PS_G10_fr3**	*Injection Volume:*	**30,0**
Vial Number:	**3**	*Channel:*	**UV_VIS_3**
Sample Type:	**unknown**	*Wavelength:*	**670**
Control Program:	**0_80_30_VIS_akl**	*Bandwidth:*	**n.a.**
Quantif. Method:	**integracja_PS**	*Dilution Factor:*	**1,0000**
Recording Time:	**2022-12-20 16:18**	*Sample Weight:*	**1,0000**
Run Time (min):	**37,00**	*Sample Amount:*	**1,0000**

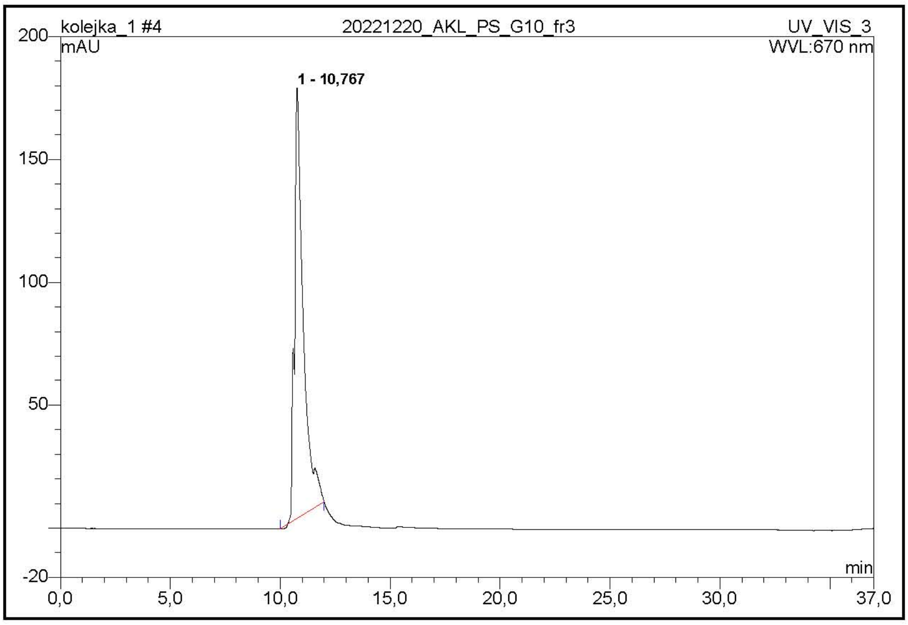

No.	Ret.Time min	Peak Name	Height mAU	Area mAU*min	Rel.Area %	Amount	Type
1	10,77	n.a.	175,183	80,156	100,00	n.a.	BMB
**Total:**			175,183	80,156	100,00	0,000	

DEFAULT/Integration

Chromeleon (c) Dionex 1996-2006
Version 6.80 SR6 Build 2491 (141932)

Figure S7. HPLC analysis of Glu-4-ZnPc using 670 nm detection.

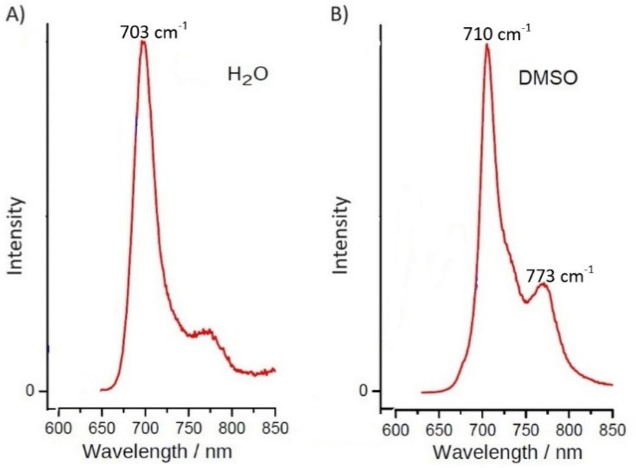

Figure S8. Emission spectra of Glu-4-ZnPc in H_2O and DMSO.

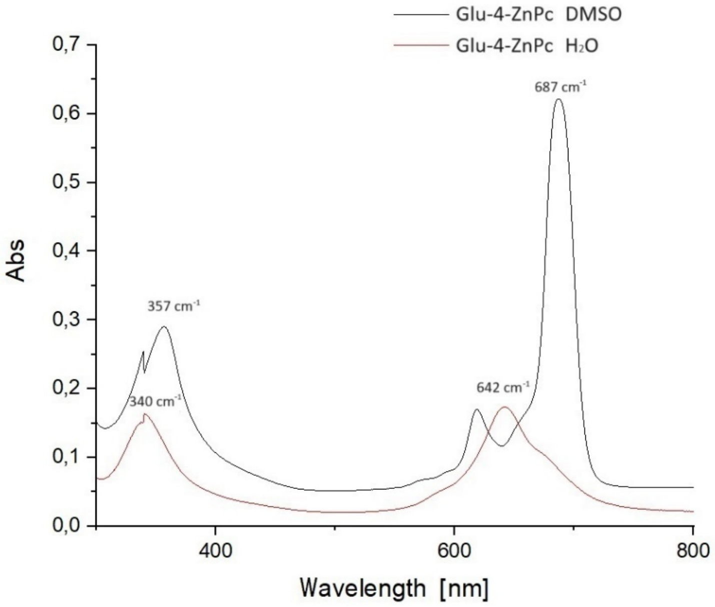

Figure S9. Excitation spectra of Glu-4-ZnPc in H_2O and DMSO.
